# Gut Microbiota and Diarrhea: An Updated Review

**DOI:** 10.3389/fcimb.2021.625210

**Published:** 2021-04-15

**Authors:** Yunxia Li, Siting Xia, Xiaohan Jiang, Can Feng, Saiming Gong, Jie Ma, Zhengfeng Fang, Jie Yin, Yulong Yin

**Affiliations:** ^1^ Animal Nutritional Genome and Germplasm Innovation Research Center, College of Animal Science and Technology, Hunan Agricultural University, Changsha, China; ^2^ Animal Nutrition Institute, Sichuan Agricultural University, Chengdu, China; ^3^ Institute of Subtropical Agriculture, Chinese Academy of Sciences, Changsha, China

**Keywords:** intestinal health, fecal microbiota transplantation, probiotics, gut microbiota, diarrhea

## Abstract

Diarrhea is a common problem to the whole world and the occurrence of diarrhea is highly associated with gut microbiota, such as bacteria, fungi, and viruses. Generally, diarrheal patients or animals are characterized by gut microbiota dysbiosis and pathogen infections may lead to diarrheal phenotypes. Of relevance, reprograming gut microbiota communities by dietary probiotics or fecal bacteria transplantation are widely introduced to treat or prevent diarrhea. In this review, we discussed the influence of the gut microbiota in the infection of diarrhea pathogens, and updated the research of reshaping the gut microbiota to prevent or treat diarrhea for the past few years. Together, gut microbiota manipulation is of great significance to the prevention and treatment of diarrhea, and further insight into the function of the gut microbiota will help to discover more anti-diarrhea probiotics.

## Introduction

Diarrhea is a common health problem in the world, which induces 1.3 million deaths every year (Troeger et al., 2017), especially for infants and young children (Lamberti et al., 2016; Black et al., 2019). Generally, diarrhea is a clinical manifestation of intestinal ion transport channel proteins, channels, physical and chemical barriers being damaged, leading to disorders of water and electrolyte transport in the digestive tract (Chu et al., 2020). In addition, diarrhea may be a symptom of many diseases. Pathological bile acid absorption, bacterial and viral infections, carbohydrate malabsorption, disaccharidase insufficiency, and chronic inflammatory diseases are all related to diarrhea (Camilleri et al., 2017). Although the mortality rate associated with diarrhea has been significantly reduced over the years, it is still one of the common reasons for pediatric emergency department visits, especially in some low-income countries in Asia and Africa (Nataro, 2013).

The intestinal tract of mammals hosts a high and diverse number of different microorganisms, including bacteria, fungi, protozoa, and viruses (Ryan et al., 2020). The density and compositions of microorganisms change along the gastrointestinal tract and perform their functions in different parts. Homeostasis and symbiotic interactions promote peaceful coexistence between the microbiota and the host, which further inhibit the colonization of most introduced pathogens and participate in nutrient absorption and physiological functions (Kaiser et al., 2012; Hillman et al., 2017; Fan and Pedersen, 2020). For example, the healthy gut microbiome can protect against epithelial cell injury and improve pathogen clearance from the gut lumen after non-typhoidal *Salmonella* diarrhea. In addition, the roles of microbial balance in the development and the maturation of the mucosal immune system and the integrity of the intestinal barrier have been also reported (Jandhyala et al., 2015). However, the compositions and diversity of gut microbiota are easily affected by various factors (i.e., diets, drugs, pathogens, and environmental factors), which further affect the health of humans and animals (Gao et al., 2013; Yin et al., 2020). A growing of evidence shows that imbalance of the gut microbiota increases the susceptibility to various pathogens and causes many diseases, including diarrhea, irritable bowel syndrome (IBS), allergies, cardiovascular disease, and obesity (Rajilić-Stojanović et al., 2015; Gresse et al., 2017; Torres-Fuentes et al., 2017).

**Figure 1:**
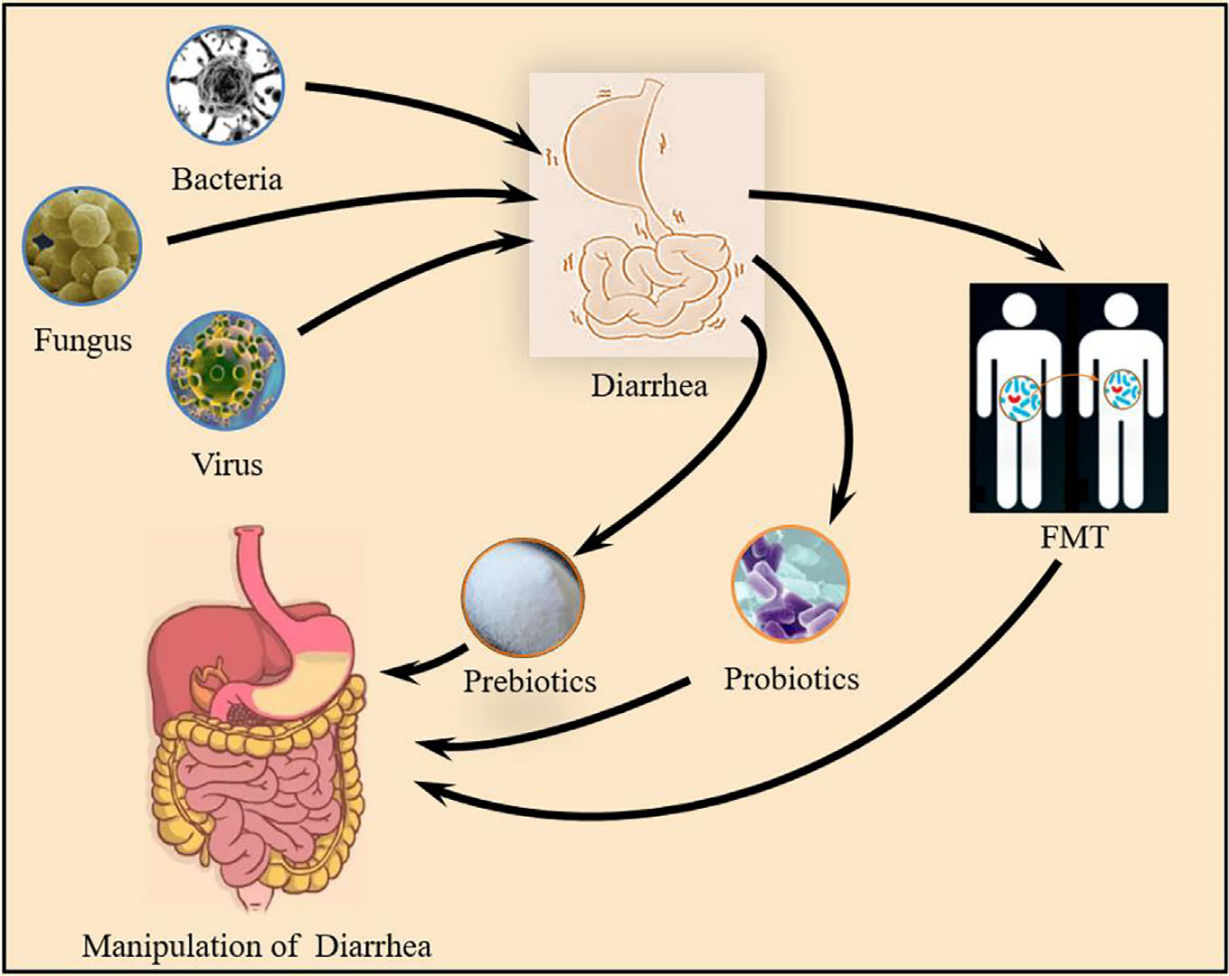
Gut microbiota and diarrhea: Bacteria, Fungus and Virus all mediate the occurrence of diarrhea. microbial intervention by prebiotics, probiotics and FMT can regulate the composition of the intestinal flora to prevent and improve the occurrence of diarrhea.

Therefore, this review aims to summarize the microorganisms that mediate the occurrence of diarrhea and combining with the newly discovered probiotics that can treat and prevent diarrhea. At the same time, the application of fecal microbiota transplantation (FMT) in the treatment of diarrhea in recent years is discussed (**Figure 1**).

## Gut Microbiota-Mediated Diarrhea and Its Mechanism

Despite an improvement of living environment conditions and vaccination, diarrhea is still a common problem faced by the whole world, especially for young children (Stockmann et al., 2017). Dysbiosis (bacteria, fungi, and viruses disorders) characterized by pathogens domination is widely identified in diarrheal humans and animals, and one area of diarrhea currently receiving a large amount of attention is the interaction with gut microbiota. Firstly, invading pathogenic bacteria inhibits the growth of normal bacteria, resulting in a decrease in the number of beneficial bacteria in the gastrointestinal tract (Fan and Pedersen, 2020). Then, pathogen-produced toxic substances further cause abnormal gut function and immune responses, leading to the occurrence of diarrhea (Ward et al., 2016). In this review, bacteria, fungi, and viruses mediated-diarrhea are mainly discussed.

### Bacteria and Diarrhea

Diarrhea caused by bacterial pathogens is a global health problem, especially in developing countries, and enteric bacterial pathogens are the main cause of infectious diarrhea. Currently, *Escherichia coli* (*E. coli*), *Shigella*, *Salmonella*, *Campylobacter*, *Clostridium difficile* (*C. difficile*), and *Aeromonas* are mainly considered to be the pathogens of diarrhea (Hodges and Gill, 2010; Kaakoush et al., 2015; Levine et al., 2020).

*E. coli* is a type of facultative anaerobic gram-negative bacteria to cause diarrhea. Different diarrheal *E. coli* strains exhibit different epidemiology and have been classified as enteropathogenic *E. coli* (EPEC, the main cause of infant diarrhea), enterohemorrhagic *E. coli* (EHEC/STEC, the cause of hemorrhagic colitis and hemolytic uremic disease), enteroaggregative *E. coli* (EAEC), enterotoxigenic *E. coli* (ETEC, the main cause of travelers’ diarrhea and infant diarrhea) and enteroinvasive *E. coli* (EIEC, the main cause of dysentery) according to the pathological types, colonization sites, virulence mechanisms, and clinical symptoms (Levine, 1987; Gomes et al., 2016). After infection, *E. coli* adheres to the intestinal epithelial cells through the adherent fimbriae, and then produces toxins and exerts pathogenic effects (Mirhoseini et al., 2018; Liu et al., 2020). Generally, diarrheal *E. coli* exhibit multi-drug resistance (Broujerdi et al., 2018), making it difficult to control the spread of pathogens.

*Salmonella*, a gram-negative and facultative anaerobic bacterium, is the third most common cause of diarrhea mortality (Troeger et al., 2017) and the infection is generally marked by gastroenteritis and diarrhea (De Jong et al., 2012). According to the clinical symptoms, *Salmonella* is divided into *Salmonella* typhi and nontyphoidal *Salmonella* (Crump et al., 2015). Typhoid *Salmonella* infections mainly occur in developing countries by causing 93.8 million food-borne cases and 155,000 deaths each year (Eng et al., 2015). While nontyphoid *Salmonella* has a wide host range (Crump et al., 2015). In addition to induce diarrhea, *Salmonella* can also cause fever and gastrointestinal complications such as pancreatitis and bleeding (Knodler and Elfenbein, 2019). Furthermore, inappropriate use of antibiotics has also led to an increase in antimicrobial resistance, and the development of multi-drug resistance in *Salmonella* serotypes has also led to an increase in the severity of *Salmonella* infections. For example, antibiotics have been found to damage the colonization resistance barrier, which further aggravates *Salmonella* infection that *Salmonella* typhimurium exceeds 10^8^ CFU/g within 24 hours of infection and causes severe intestinal diseases (Ackermann et al., 2008). This epidemiological evidence underscores the relevance of these studies on mice to human diseases, that is, humans are more susceptible to *Salmonella* gastroenteritis after antibiotic treatment.

*C. difficile* is a gram-positive anaerobic bacterium with capable of forming a spore structure, which is widely present in the intestines of humans and animals. *C. difficile* infection has become the main cause of antibiotic-related diarrhea worldwide (Peng et al., 2018; Mileto et al., 2020). The clinical symptoms of *C. difficile* infection are ranging from asymptomatic carriers to various degrees of diarrhea even death. When the normal gut microbiota community is destroyed, *C. difficile* begins to colonize and dominate in the large intestine, releasing enterotoxin A and cytotoxin B (Androga et al., 2015). These toxins further damage the epithelial cells cytoskeleton, causing severe intestinal inflammation, diarrhea, and pseudomembranous colitis (Czepiel et al., 2019). Meanwhile, *C. difficile* may produce indole by manipulating the local microbial community, which has an effect on the abundance and diversity of bacterial communities in the colon, inhibiting the growth of beneficial indole-sensitive bacteria, such as *Bacteroides* and *Edwardsiella* (Darkoh et al., 2019). With the extensive use of broad-spectrum antibiotics, the resistance of strains has increased and highly virulent strains have emerged, which significantly increases the incidence of *C. difficile* infection. In mouse models, it was found that the isolated *C. difficile* variants from outbreaks of *C. difficile* – associated disease induced a strong inflammatory response (McDonald et al., 2005; Quesada-Gómez et al., 2015).

*Shigella* is a form of gram-negative bacterium to cause animal and human diarrhea (Liu et al., 2019). It is estimated that *Shigella* causes approximately 125 million diarrhea episodes and approximately 160,000 deaths every years, with a third are related to young children (Baker and The, 2018). *Shigella* produces *Shigella* enterotoxin and serotype toxin 1 into the enteric cavity, which further invade and destroy the epithelium of the large intestine, eventually leading to aggressive watery or mucus-like/bloody diarrhea (Nisa et al., 2020).

*Vibrio cholerae* is a gram-negative bacterium that causes watery diarrhea in the host. The cholera toxins, adnexa cholera toxin and closed band toxin produced by *V. cholerae* cause activation of anion secretion, inhibit absorption of electroneutral NaCl, destroy intestinal barrier function, and cause severe diarrhea (Ramamurthy et al., 2020). The serotypes O1 and O139 of *V. cholerae* strains with two main virulence genomes: cholera toxin (CT) and pathogenic toxin colonies cause an acute watery diarrhea (Ogura et al., 2020). A recent study showed that some non-01/O139 *V. cholerae* (NOVC) strains also cause diarrhea (Vezzulli et al., 2020). *V. cholerae* can also affect the virulence of pathogenic *Escherichia coli*, and the virulence of EPEC is enhanced with the elevated concentration of cholera autoinducer 1 (CAT-1) when grown in co-culture with *V. cholerae* (Gorelik et al., 2019).

### Fungus and Diarrhea

Fungi are an important part of gut microorganisms and certain fungal communities have been confirmed to be highly associated with diarrhea (Sangster et al., 2016). *Candida* is generally considered to be a reliable cause of diarrhea, but its mechanism of inducing diarrhea is still unclear (Awoyeni et al., 2017). It is generally believed that *Candida* may selectively cause diarrhea in a clinical setting (Ponnuvel et al., 1996). *Candida albicans* (*C. albicans*), a conditional pathogen, is the most abundant fungus in the intestine of mammals (Nobile and Johnson, 2015). The role of C. albicans and diarrhea has been controversial for many years. While in mouse model, it was found that *C. albicans* could cause intestinal dysbiosis and enhance the severity of DSS-induced colitis (Panpetch et al., 2020). The mechanisms may be associated with dectin-1, which is a C-type lectin-like receptor that mediates the fungal immune response. Dectin-1 expression is positively correlated with (1,3)-β-Glucan and mediates fungal infections by recognizing the (1,3)-β-glucan structure on the fungal cell wall (Gantner et al., 2005). In addition, *Candida krusei*, *Candida tropicalis*, *Candida glabrata*, *Candida guilliermondii*, *Candida parapsilosis* are also the main pathogens that cause invasive candidiasis that may be implicated in diarrhea, but the detailed mechanisms need to be further studied (Awoyeni et al., 2017; Hirayama et al., 2020).

### Virus and Diarrhea

The viral microbiome is a complex community composed of eukaryotic RNA viruses, DNA viruses, and bacteriophages, which plays an important role in maintaining human and animal health. Previous studies showed that some viruses also contribute to diarrhea in humans and animals and the clinical symptoms of viral diarrhea include diarrhea, fever, and vomiting (Goodgame, 2001). Rotavirus is a common diarrheal pathogen in infants and young animals, causing more than 200,000 deaths every year (Ramig, 2004). Rotavirus, a double RNA virus without envelope, infects intestinal epithelial cells to stimulate intestinal secretion and activation of the enteric nervous system, causing the destruction of absorptive intestinal epithelial cells, thereby inducing diarrhea (Tafazoli et al., 2001; Crawford et al., 2017). Reduction of absorption function of the intestinal epithelium by rotavirus infection has been widely reported to cause damage and death of intestinal cells *in vivo* and vitro (Ball et al., 1996; Morris et al., 1999). In addition, viruses that cause diarrhea also include Norovirus, Astrovirus, Enterovirus, and Boca virus, while Rotavirus infection is usually more serious compared with other sources of infection (Crawford et al., 2017).

## Manipulation of Diarrhea by Reprograming Gut Microbiota

Gut microbiota plays a great significance to intestinal health, and healthy gut microbiota can resist the colonization of diarrhea pathogens. It was found in germ-free mice and antibiotic-treated mice that the gut microbiota has a protective effect on diarrhea infection (Kamada et al., 2012; Vogt and Finlay, 2017). Modulating the gut microbiota to improve human health also become more and more important. Diet is an important modulator of the gut microbiota, that can regulate the composition and function of the community of microbes in humans and other mammals to resist diseases (Sonnenburg and Bäckhed, 2016). In past studies, it was also found that microbial intervention can regulate the composition of the intestinal flora to prevent and improve the occurrence of diarrhea (Gallo et al., 2016).

### Probiotics and Diarrhea

Probiotics are considered to be beneficial to the host’s health and contain a sufficient amount of non-pathogenic specific live bacteria preparations, such as *Lactobacillus*, *Yeast*, *Bifidobacterium*, *Enterococcus*, and *Bacillus*. Probiotics have been widely reported to treat pathogens-caused diarrhea by maintaining or improving the balance of gut microbiota and the mechanisms may be associated with the inhibitory effect on the colonization of harmful bacteria by competing for nutrients and producing antibacterial compounds (Bron et al., 2017). Probiotics can reduce the severity of *Citrobacter rodentium*, *Listeria monocytogenes*, EHEC, and *Salmonella typhimurium* infections (Bayoumi and Griffiths, 2010; Emara et al., 2016; Wen et al., 2019). For example, *Bifidobacterium breve* and *Bifidobacterium pseudocatenulatum* DSM20439 inhibit the expression of intestinal Shiga toxin EHEC (Asahara et al., 2004). In clinic, the incidence of antibiotic-associated diarrhea and *C. difficile*-associated diarrhea in patients who received probiotic capsules per day was lower than that in the placebo group (Gao et al., 2010). Similarly, oral Lactobacillus LGG reduced the incidence of diarrhea in children and shorten the course of diarrhea (Li et al., 2019). Similarly, the administration of fortified milk containing probiotic Bifidobacterium lactis HN019 (1.9×10^7^CFU) and prebiotic oligosaccharides administered three times a day for a year can reduce the incidence of dysentery in children (Sazawal et al., 2010). In addition, Szajewska and Mrukowicz reported that probiotics reduced the duration of Rotavirus diarrhea (Szajewska and Mrukowicz, 2001). Other studies further confirmed that probiotics such as *Bifidobacterium* and *Lactobacilli* played an important role in the treatment of Rotavirus infection (Rigo-Adrover et al., 2018; Azagra-Boronat et al., 2020). For example, *Lactobacillus rhamnosus* GG regulates the maturation and differentiation of dendritic cells (DCs) and the secretion of inflammatory factors, thereby preventing diarrhea caused by Rotavirus (Jiang et al., 2017). Together, the beneficial effects of probiotics on diarrhea are related to the strain and dosage and the selection and use of the best probiotics for the treatment of diarrhea need to be determined by more clinical trials.

The anti-diarrheal mechanisms of probiotics have not yet been fully elucidated, the currently considered mechanisms of action of probiotics mainly rely on the following pathways: (1) regulation of the balance of gut microbiota; (2) improvement immunity; (3) manipulation of intestinal defense barrier (Monteagudo-Mera et al., 2019); (4) metabolites. Firstly, probiotics have been widely considered to maintain gut microbial hemostasis and a healthy gut microbiota community predicate a low incidence of diarrhea. In addition, probiotics can produce organic acids in the metabolic process and lower the pH of the gut cavity, thereby inhibiting the growth of pathogens (Peters et al., 2019). Secondly, probiotic strains serve as a key activator for the gut innate and adaptive immune systems, thereby signaling antimicrobial and inflammatory responses and enhancing the diarrheal resistance (Kawashima et al., 2018). Thirdly, probiotics can stimulate the production and secretion of mucin, cathelicidins, and defensins from goblet cells and epithelial cells to form an immune barrier, preventing the invasion of pathogenic bacteria (Do Carmo et al., 2018). Meanwhile, probiotics have been reported to increase the expressions of tight junction proteins and reduce the damage to the gut tissues caused by pathogenic bacteria such as *E. coli* and Rotavirus (Zeng et al., 2017; Paparo et al., 2020). More interestingly, Hu Jun et al. transplanted fecal microbiota between CM piglets (a native Chinese breed, are more resistant to early-weaning stress-induced diarrhea) and commercial crossbred LY piglets and identified *Lactobacillus gasseri* LA39 (*L. gasseri* LA39) and *Lactobacillus frumenti* (*L. frumenti*) that could mediate diarrhea resistance. The mechanisms may be associated with microbiota-derived bacteriocin gasericin A, which further binds to Keratin 19 on the plasma membrane of intestinal epithelial cells to regulate fluid absorption and secretion (Hu et al., 2018). Recent studies have reported that adjusting the abundance and type of intestinal bacteria through *Debaryomyces hansenii* treatment can alleviate diarrhea caused by antibiotics. The reason may be that *Debaryomyces hansenii* changed the bacterial community structure, and promoted the growth of some key lactase-producing bacteria, such as *Enterobacter* sp. *638* and *Modestobacter* (He et al., 2017; Wu et al., 2020). Intestinal lactase is mainly produced by microorganisms such as *Lactobacillus* sp., *Bacillus* sp., *Escherichia coli*, *Bifidobacterium* sp., *Enterobacter aerogenes* and *Streptococcus thermophilus*, low activity and lactase deficiency can cause diarrhea (Long et al., 2017). Summarily, probiotics not only inhibit the overgrowth of pathogens, but also enhance the anti-pathogenic ability against microbiota associated with diarrhea.

### Prebiotics and Diarrhea

Prebiotics are defined as “substrates that are selectively utilized by host microorganisms to confer health benefits” (Gibson et al., 2017). Consumption of prebiotics can improve the gut microbiota, which is beneficial to health. Recent study has shown that interventions prebiotic can increase the production of SCFA, play an important role in maintaining the intestinal barrier (Azad MAK et al., 2020; Snelson et al., 2021). The gut lymphoid tissue can induce secrete cytokines and anti-microbial peptides, such as β-defensins, to defense against the invasion of microorganisms (De Santis et al., 2015). Some prebiotics, such as fructo oligosaccharide, inulin, pectin oligosaccharides, etc., can resist the colonization of pathogen by acting as soluble decoy receptors that mimic the binding site of pathogens, thereby promoting the eliminating of pathogens from the intestine (Pujari and Banerjee, 2021). Previous studies have shown that prebiotics can shorten the duration of acute watery diarrhea and has a good therapeutic effect on diarrhea (Rigo-Adrover et al., 2017).

### FMT and Diarrhea

FMT has become the focus of research in biomedicine and clinical medicine in recent years. FMT refers to the process of transplanting the functional flora in the feces of healthy people into the gastrointestinal tract of the patient to rebuild the gut flora with normal functions to treat intestinal and extra-intestinal diseases (De Groot et al., 2017). The clinical response of FMT to different diseases (i.e., Clostridium difficile infection, inflammatory bowel disease, diabetes, cancer, liver cirrhosis, and brain diseases) provides direct evidence for the interaction between the microbiota and the host (Aron-Wisnewsky et al., 2019; Tariq et al., 2020; Zhang et al., 2020). In a randomized double-blind trial, patients received anaerobic prepared donor FMT relieved ulcerative colitis (Costello et al., 2019). In another study, FMT significantly alleviated symptoms in IBS patients by double-blind randomized trials, but there have also been contradictory results (Halkjaer et al., 2018; Johnsen et al., 2018). In dextran sodium sulfate (DSS)-induced murine colitis model, fecal microorganisms from normal biological donors reduced colitis by regulating the expression of pro-inflammatory genes, antibacterial peptides and mucins (Burrello et al., 2019). In another two studies, the results showed that FMT increased the number of beneficial bacteria in the intestinal tract and reduced the number of harmful bacteria, and further research showed that FMT triggered the intestinal mucosal autophagy and reduce the damage of the intestinal barrier caused by *E. coli K88* (Cheng et al., 2018).

The therapeutic effect of FMT in gut diseases is related to reprogram gut microbiome, and the healthy flora reintroduced through FMT will compete with the ecological environment and prevent the colonization of pathogens. One way for the gut microbiota to directly inhibit the pathogen of diarrhea is to compete for nutrients to reject the pathogen (Vogt and Finlay, 2017). At the same time, the gut microbiota secrete antibacterial compounds that further inhibit the growth of harmful bacteria, such as bacteriocins or small molecular metabolites (Antunes et al., 2014; McKenney and Pamer, 2015). For example, some symbiotic bacteria can produce short-chain fatty acids (SFCAs), which can change the local pH to inhibit the growth of pathogens (Shin et al., 2002). However, the research on the mechanisms of FMT is limited, and it is not clear whether the changes of these microbiota have played a sufficiently important role. In recent studies, it has been proposed that the role of FMT in disease may not only be explained by simply restoring intestinal bacteria itself (Ott et al., 2017). Therefore, the application of FMT in diarrhea related diseases and the function of FMT in recipients need further studies.

## Conclusion

Diarrhea is related to changes of gut microbiota and balanced gut microbiota is resistant to the colonization of diarrhea pathogens. Based on bacterial therapy, the gastrointestinal tract constitutes a healthy microbiota throughout life, which plays an important role in the prevention and treatment of diarrhea. Although the specific mechanism of gut microbiota affecting diarrhea remains to be studied in depth, it is effective to prevent and treat diarrhea by regulating the gut microbiota by dietary probiotics and FMT. Despite the progress made in the understanding gut microbiota and diarrhea, there are a number of prominent research avenues remain to be explored. For example, diarrhea is highly associated with gut microbiota alterations, thus microbial quorum sensing during the pathogen infection should be studied to unveil the pathogenesis of diarrhea; In addition, different probiotics exhibit different probiotic properties, the detailed mechanisms of probiotics-mediated diarrheal pathogen clearance are suggested; Nutritional regulation has been widely introduced to manipulation of gut microbiota compositions, personalized diets are designed to improve gut microbiota compositions against pathogen infections; Currently, FMT has been widely recommended to treat gut microbiota-related diseases, donor selection, ethics, operation method, and the follow-up should be standardized to guarantee the safe and effective of FMT in clinical practice. Further studies believe that the research on the function of microflora will help to discover some potential probiotics with anti-diarrhea function.

## Author Contributions

YL: Writing-Original draft preparation, revision and investigation. JY, SX and JM: Revision. XJ, CF and SG: Conceptualization. ZF: investigation. YY: Supervision. JY: Validation. All authors contributed to the article and approved the submitted version.

## Funding

This study was supported by the Key Programs of frontier scientific research of the Chinese Academy of Sciences (QYZDY-SSW-SMC008), Young Elite Scientists Sponsorship Program by CAST (2019-2021QNRC001), and Hunan Science Foundation for Outstanding Young Scholars (2020JJ3023).

## Conflict of Interest

The authors declare that the research was conducted in the absence of any commercial or financial relationships that could be construed as a potential conflict of interest.
